# Single-cell RNA sequencing analysis of lung cells in COVID-19 patients with diabetes, hypertension, and comorbid diabetes-hypertension

**DOI:** 10.3389/fendo.2023.1258646

**Published:** 2023-12-08

**Authors:** Xin Zhang, Xiaoqian Deng, Liangliang Zhang, Pengbo Wang, Xia Tong, Yan Mo, Yuansheng Zhang, Yan Zhang, Chunheng Mo, Lanlan Zhang

**Affiliations:** ^1^ Department of Pulmonary and Critical Care Medicine, State Key Laboratory of Respiratory Health and Multimorbidity, West China Hospital, Sichuan University, Chengdu, China; ^2^ Department of Gastroenterology, West China (Airport) Hospital of Sichuan University (The First People’s Hospital of Shuangliu District, Chengdu), Chengdu, China; ^3^ Department of Anesthesiology, West China Hospital, Sichuan University, Chengdu, China; ^4^ School of Professional Studies, Columbia University, New York, NY, United States; ^5^ Department of Neurology Medicine, The Aviation Industry Corporation of China (AVIC) 363 Hospital, Chengdu, China; ^6^ Department of Gastroenterology, West China Hospital, Sichuan University, Chengdu, China; ^7^ Key Laboratory of Birth Defects and Related Diseases of Women and Children of MOE, West China Second University Hospital, Sichuan University, Chengdu, China; ^8^ State Key Laboratory of Biotherapy, West China Second University Hospital, Sichuan University, Chengdu, China

**Keywords:** SARS-CoV-2, diabetes, hypertension, endothelial cells, fibroblasts

## Abstract

**Background:**

There is growing evidence that the lung is a target organ for injury in diabetes and hypertension. There are no studies on the status of the lungs, especially cellular subpopulations, and related functions in patients with diabetes, hypertension, and hypertension-diabetes after combined SARS-CoV-2 infection.

**Method:**

Using single-cell meta-analysis in combination with bulk-RNA analysis, we identified three drug targets and potential receptors for SARS-CoV-2 infection in lung tissues from patients with diabetes, hypertension, and hypertension-diabetes, referred to as “co-morbid” patients. Using single-cell meta-analysis analysis in combination with bulk-RNA, we identified drug targets and potential receptors for SARS-CoV-2 infection in the three co-morbidities.

**Results:**

The single-cell meta-analysis of lung samples from SARS-CoV-2-infected individuals with diabetes, hypertension, and hypertension-diabetes comorbidity revealed an upregulation of fibroblast subpopulations in these disease conditions associated with a predictive decrease in lung function. To further investigate the response of fibroblasts to therapeutic targets in hypertension and diabetes, we analyzed 35 upregulated targets in both diabetes and hypertension. Interestingly, among these targets, five specific genes were upregulated in fibroblasts, suggesting their potential association with enhanced activation of endothelial cells. Furthermore, our investigation into the underlying mechanisms driving fibroblast upregulation indicated that KREMEN1, rather than ACE2, could be the receptor responsible for fibroblast activation. This finding adds novel insights into the molecular processes involved in fibroblast modulation in the context of SARS-CoV-2 infection within these comorbid conditions. Lastly, we compared the efficacy of Pirfenidone and Nintedanib as therapeutic interventions targeting fibroblasts prone to pulmonary fibrosis. Our findings suggest that Nintedanib may be a more suitable treatment option for COVID-19 patients with diabetes and hypertension who exhibit fibrotic lung lesions.

**Conclusion:**

In the context of SARS-CoV-2 infections, diabetes, hypertension, and their coexistence predominantly lead to myofibroblast proliferation. This phenomenon could be attributed to the upregulation of activated endothelial cells. Moreover, it is noteworthy that therapeutic interventions targeting hypertension-diabetes demonstrate superior efficacy. Regarding treating fibrotic lung conditions, Nintedanib is a more compelling therapeutic option.

## 1 Introduction

Diabetes and hypertension are common microvascular and macrovascular diseases that affect multiple organs. The alveolar-capillary network in the lungs is a large microvascular unit that may be affected by microvascular pathology (1–7). Considering the microvascular effects of diabetes and hypertension in the retina and glomeruli, the microvasculature in the lungs may also be affected by them (8). However, because the lungs have a large reserve capacity, much of the loss from microvascular damage can be tolerated without the need for symptoms of dyspnea. Therefore, microvascular pathology due to diabetes in the lungs may be underestimated, especially when accompanied by reduced lung function. Loss of posterior elasticity produced by collagen glycosylation in lung tissue has been suggested as a possible mechanism leading to this condition (9). There is a correlation between insulin resistance and hypoxia-induced by low birth weight with insulin resistance and impaired lung function, which may be related to collagen glycosylation in lung tissue. Several epidemiologic and clinical studies have found that adults with diabetes have an increased risk of decreased lung function compared to adults without diabetes (10, 11). However, studies on the exact relationship between decreased lung function and diabetic hypertension, as well as the pathophysiologic mechanisms, have not reached a consensus conclusion.

Current efforts following coronavirus disease 2019 (COVID-19) infection focus on new crown sequelae (12–14). COVID-19 exhibits similar physiological responses and clinical features in patients with diabetes and hypertension. However, each individual is involved in a different molecular pathway before or during SARS-CoV-2 infection. Several potential pathways have been proposed, including increased inflammatory storms (15), immunocompromised state, dysfunctional glucose homeostasis, hypercoagulability, alveolar hyperpermeability and vascular endothelial damage (9). Activation of these molecular pathways determines whether new-onset diabetic symptoms and complications are temporary or persist after viral clearance. However, to date, no study has given a definitive answer as to the effect of the superimposition of these three diseases on the sequelae of lung tissue, such as fibrosis.

On the medication of COVID-19 in diabetes combined with hypertension, GLP-1R agonists, DPP-4 inhibitors, or pioglitazone were associated with significant reductions in hospital admissions, respiratory complications, and mortality, and may improve COVID-19 outcomes in patients with T2DM (16). Hypertensive patients treated with long-term ACE inhibitors or ARBs have a lower risk of COVID-19 compared with CCBs. These results, if confirmed, often contradict previous hypotheses and suggest new ones (17). However, a fibrotic complication such as pulmonary fibrosis caused by COVID-19 patients, which is a serious prognostic threat, also deserves our attention (18–23). Direct evidence that SARS-CoV-2 causes pulmonary fibrosis: pulmonary fibrosis was found in autopsy and lung puncture pathology. Indirect evidence: transforming growth factor TGF-β, tumor necrosis factor TNF-α, and interleukin IL-6 were elevated in lung tissues, and the therapeutic value of two drugs (Pirfenidone and Nintedanib) for idiopathic pulmonary fibrosis in COVID-19-induced pulmonary fibrosis. However, it has not been analyzed from a molecular point of view whether fibrosis caused by patients with “co-morbidities” is effective in the treatment with Pirfenidone and Nintedanib.

The process of lung tissue subpopulation change cannot be adequately captured by clinical or molecular assays alone, and thus a comprehensive systems biology strategy is needed to address the complexity of this multiple cellular state change in order to unravel the mechanisms involved in new-onset diabetes. Single-cell sequencing is a technological approach to assessing cellular function in lung tissue, and by performing single-cell analyses in COVID-19 patients with comorbid diabetes mellitus, hypertension, and hypertension-diabetes, we sought to answer the following questions:1. which cellular subpopulations undergo the greatest changes after comorbid co-morbidities? 2. what are the underlying mechanisms of these changes? 3. which cellular subpopulations are predictive of lower lung function? 4. which diabetes and hypertension medications are more effective? 5. Pirfenidone and Nintedanib, which are more effective in comorbidity-induced pulmonary fibrosis? To answer the above questions, we performed single cell RNA sequencing combined bulk-RNA analysis aimed at supporting clinical diagnosis and treatment from a single-cell perspective.

## 2 Methods

### 2.1 Data collection

#### 2.1.1 Single cell RNA sequencing datasets

We downloaded the available COVID-19 and IPF-related scRNA-seq datasets from Gene Expression Omnibus (GEO) and collected the datasets as follows:

1) COVID-19: The characteristics of the included studies in our article were as follows: 1) Patients with confirmed diagnoses of COVID-19. 2) Human lung tissue samples. 3) All lung cell types. 4) Availability of clinical information regarding comorbidities such as hypertension and diabetes. The datasets included in our analysis were GSE171524, GSE171668, GSE149878, GSE161382, and GSE163919. These datasets encompassed a total of 131,887 cells from 45 healthy controls, 45,473 cells from 10 patients with COVID-19, 6,948 cells from 1 patient with COVID-19 combined with diabetes, 106,618 cells from 22 patients with COVID-19 combined with hypertension, and 81,810 cells from 22 patients with COVID-19 combined with both hypertension and diabetes (**Table S1**).

2) Idiopathic pulmonary fibrosis (IPF): GSE132771, GSE135893, GSE122960, GSE128033, GSE128169, GSE136831, GSE159354.These 7 datasets included 77 Control, 87 IPF, 15 systemic sclerosis (SSc), 4 chronic hypersensitivity pneumonitis (cHP), 1 Myositis, 4 non-specific interstitial pneumonia (NSIP), 3 Sarcoidosis. (**Table S2**).

#### 2.1.2 Bulk RNA datasets

GSE47460, 254 frozen tissue samples from interstitial lung diseases (ILD) patients were collected from GEO.

### 2.2 Flow of single-cell sequencing data analysis

#### 2.2.1 scRNA/snRNA data processing

We used Seurat package (v4.0) to analyze scRNA/snRNA sequencing data. The specific steps are as follows: 1) Data quality control: remove low-quality cells with gene number less than 200 or more than 5000 and remove cells with more than 20% of mitochondrial genes. 2) Data normalization: execute LogNormalize, FindVariableFeatures, ScaleData functions, respectively. 3) Use Harmony Perform sample integration and batch effect removal. 4) Downscaling and visualization: use RunPCA function for principal component analysis, FindNeighbors for clustering, FindClusters for cell subpopulation analysis, and UMAP for dimensional reduction. 5) **Cell type identification:**cell types were determined mainly by “ FindAllMarkers”, classical cell marker genes, and prediction using R packages (clustermole, singscore) is done jointly.

#### 2.2.2 Identification and functional analysis of differentially expressed genes

(1) **Identification of DEGs**: the FindMarkers function was used to find DEGs between different cell subpopulations or between different diseases of the same cell subpopulation. Here, we defined that a P-value less than 0.05 and avg_log_2_FC>0.25 were statistically significant DEGs.
(2) **Functional analysis of gene clusters**: Use the “clusterProfiler” package in R to analyze the GO/KEGG enrichment of DEGs of cell subpopulations, and further determine their contribution to biological functions.
(3) **Gene set scoring**: AUCell was used to score the enriched relevant signaling pathways on individual cells, the scores were differentiated by different color scales, where yellow scored high and black scored low. As well as using the Seurat toolkit AddModuleScore function to calculate the scores of genes and gene sets on individual cells, the scores are differentiated by different color scales, with purple scoring high and gray scoring low in the Addmodule.

### 2.3 Integration of scRNA/snRNA dataset and dataset to identify features associated with low lung function in patients with pulmonary fibrosis

Expression matrices of bulk-RNA GSE47460 data and clinical phenotypes of lung function were used. single-cell expression matrices of COVID-19 patient lung tissue fibroblasts and endothelial cells were used, respectively. The expression matrix of GSE47460 data and the clinical phenotype lung function were integrated with the cellular dataset using the Scissor algorithm to identify cellular subpopulations or disease subtypes associated with the clinical phenotype lung function. Cell subpopulations of Scissor+, Scissor- were identified. Differential genes for Scissor+ and Scissor- cellular subpopulations were obtained using the FindMarkers function (p-values less than 0.05 and avg_log_2_FC > 0.25 were statistically significant DEGs).

### 2.4 Selection of drug target genes and gene-positive cell extraction

Hypertensive drug, diabetic drug, Nintedanib and Pirfenidone target genes were selected based on literature reports. Among the hypertensive drug target genes were Calcium channel blockers (CCBs):KCNMB1, CACNA1C, CACNB2, CACNA1D, CYP3A5, CYP3A4, ABCB1; Angiotensin-II Receptor Blockers (ARB) and Angiotensin-Converting Enzyme Inhibitors (ACEi): NPHS1, NOS3, CAMK1D, SCNN1G, GPR83, PRKCA, BDKRB2; Polymorphisms in Genes Affecting the Adrenergic Receptor Blocker Response : ADRB1, GRK4, FGD5, SLC25A31, ACY3; Polymorphisms in Genes Affecting Diuretic Response: ACE, ADD1, GNB3. ALDH1A2, LYZ, YEATS4, FRS2, NEDD4L. Diabetes drug target genes include: Glucagon-like peptide-1 (GLP-1): GLP1R; Novel glucose-sodium co-transport protein 2 (SGLT2). SLC5A2; Dipeptidyl peptidase IV (DPP-IV): DPP4; Peroxisome proliferator-activated receptor (PPAR): PPARG, PPARA, PPARB; Protein tyrosine phosphatase-1B (PTP-1B): PTPN1; Glucokinase agonists (GKA): GCK; 11β-hydroxysteroid dehydrogenase type1 (11β-HSDl) inhibitors, including HSD11B1. Nintedanib target genes are: PDGFRB, PDGFRA, KDR, FLT4, FLT1, FGFR3, FGFR2, FGFR1. Pirfenidone target genes were TGFB1, TGFB2,TGFB3,TGFBR1,TGFBR2; FGF2; MMP2, TIMP1, SERPINH1.

The above target gene-positive cells were extracted, and the proportion of target gene-positive cells in each cell subpopulation of different types of patients was calculated for further Meta analyses and functional analyses.

### 2.5 Meta analyses

Meta-analysis was done separately using the continuous variable metacount function of the meta data package of R software. The overall difference in means between different patients and healthy controls was quantified by pooling the differences provided by the original studies using a random effects model, and the results are presented as forest plots. Results for the proportion of gene-positive cells are given as mean difference MD (95% CI). Statistical heterogeneity between studies was assessed using I². For results from more than 2 studies, publication bias was assessed using Egger regression.

### 2.6 Analysis of intercellular communication

Cell communication analysis between activated endothelial cells and myofibroblasts was performed using the NicheNet toolkit to predict ligand-receptor connections between the two cells.

Communication analysis between myofibroblasts and the rest of the cells was performed using CellChat, which uses network analysis and pattern recognition methods to predict the major signaling inputs and outputs of the cells, as well as the probability of communication of ligand-receptor pairs between these cells.

### 2.7 Statistical methods

All data are expressed as mean ± standard error. Comparisons between groups were performed using t-tests, and comparisons between multiple groups were performed using one-way ANOVA. All calculations or analyses were performed using the Prism 9 software package (GraphPad) or R language. DEGs in each cluster were analyzed using the Wilcoxon rank sum statistical test. *Correlations* between two variables were analyzed using Pearson correlation analysis, and P<0.05 was considered statistically significant.

## 3 Results

### 3.1 Fibroblasts have worse lung function in all three disease

**Figure 1:**
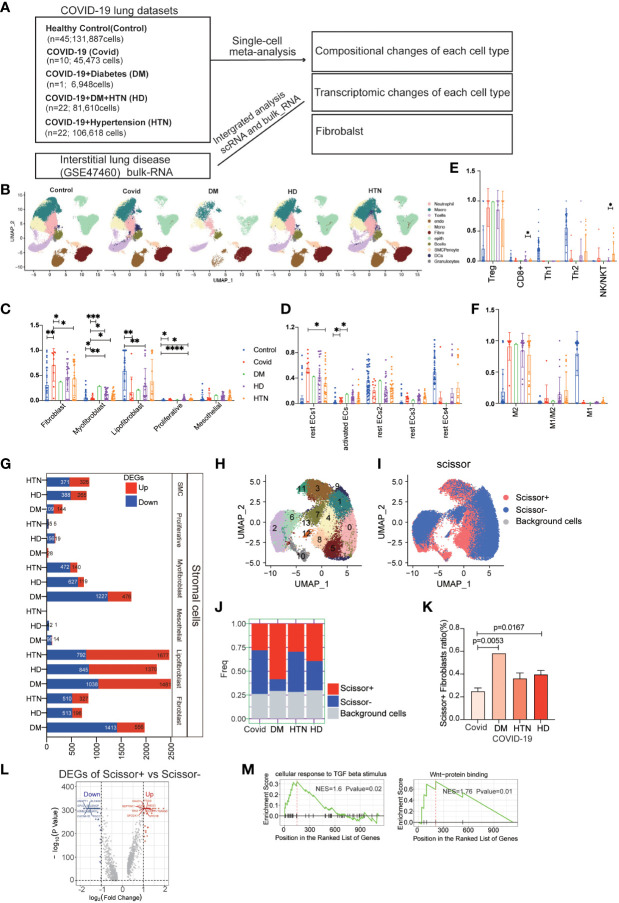
COVID-19 patients with combined diabetes/hypertension showed large differences in the number and transcriptome levels of lung cell subpopulations and were more prone to pulmonary fibrosis. **(A)** The lung scRNA-seq and bulk-RNA datasets used in the meta-analysis consisted of 131,887 cells from 45 healthy controls, 44,316 cells from 10 COVID-19 patients, 6,717 cells from 1 COVID-19 patient with diabetes, 79,290 cells from 22 COVID-19 patients with hypertension, and 103,911 cells from 22 COVID-19 patients with both hypertension and diabetes. Additionally, bulk RNA data from 254 frozen tissue samples from ILD patients were obtained from the GSE47460 dataset. **(B)** UMAP visualization of all cell types (Fibroblasts, T cells, Macrophages, Endothelials, Epithelials, Neutrophils, Plasma, Monocytes, Ciliated, SMC, Granulocytes, B cells, Mast, DCs) in the Control, DM, HTN, and HD groups, with cell types color-coded. C-F. Bar graphs displaying significant differences in the proportions of cell subtypes in the Covid, DM, HTN, and HD groups compared to the Control group in fibroblast **(C)**, endothelial **(D)**, T cell **(E)**, and macrophage **(F)** samples from lung tissues. The proportions of fibroblast cells were significantly higher in the DM, HTN, and HD groups compared to the Control group, as shown in Figure **(C)** Compared to the healthy control group, myofibroblasts were increased in the Covid, DM, HD, and HTN groups, with a statistically significant difference in the Covid group. Relative to the Covid group, myofibroblasts showed significant upregulation in both the DM, HD, and HTN groups, as illustrated in Figure **(C)** Regarding endothelial cells, the proportion of activated endothelial cells (activated ECs) was higher in the Covid, DM, HTN, and HD groups, with a statistically significant difference in the Covid group. Relative to the Covid group, activated ECs showed upregulation in both the DM, HD, and HTN groups, as illustrated in Figure **(D)**. **(G)** Bar graph exhibiting the number of differentially expressed genes (DEGs) in fibroblasts from COVID-19 patients, comparing the DM, HTN, HD, and Covid groups, categorized by cell type (FDR < 0.05). **(H)** UMAP visualization of fibroblasts in the four groups, with clusters color-coded. **(I)** Scissor analysis relating phenotypic data of lung function from bulk-RNA data of IPF (idiopathic pulmonary fibrosis) patients from the GEO dataset with scRNA-seq data of COVID-19 patients. UMAP plots depict the position of cells associated with normal lung function (blue) or low lung function (red). **(J, K)** Bar graph displaying the proportion of Scissor+ fibroblast cells in the four disease groups. The proportion of Scissor+ fibroblast cells was significantly higher in the DM, HTN, and HD groups compared to the Control group, with statistically significant differences observed in the DM and HD groups. **(L)** Volcano plot exhibiting the differentially expressed genes (DEGs) between Scissor+ and Scissor- cells. It shows the number of upregulated and downregulated genes. **(M)** GSEA enrichment analysis of DEGs in Scissor+ and Scissor- cells, indicating that the upregulated DEGs were significantly enriched in TGF-beta and Wnt-related signaling pathways. The NES (normalized enrichment score) represents the statistical score of the enriched pathway, with positive scores signifying enrichment at the front of the sorted sequence, and negative scores indicating enrichment at the back. The P value indicates the statistical significance of the enrichment score for a pathway gene set, where smaller values indicate better enrichment.

Single-cell sequencing data were obtained from public databases, encompassing pulmonary SARS-CoV-2 infection cases in 10 COVID-19 patients (abbreviated as Covid). In addition, data from 1 COVID-19 patient with concurrent diabetes mellitus (abbreviated as DM), 22 COVID-19 patients with concurrent hypertension (abbreviated as HTN), and 22 COVID-19 patients with combined hypertension and diabetes mellitus (abbreviated as HD), 54 Healthy patients (Control) were included. To analyze the bulk RNA (bulk-RNA) data and identify the disease associated with lower lung function, we employed single-cell subpopulation analysis and incorporated indicators of lung function (**Figure 1A**, **sFigure 1A**). Through the application of cellular subpopulation clustering, we identified a total of 16 distinct subpopulations (**Figure 1B**). By extracting the subpopulation ratios for each sample, we conducted statistical analysis, which revealed notable alterations in the fibroblast subpopulations. Compared to the healthy control group, myofibroblasts were significantly increased in the Covid, DM, HD, and HTN groups. Relative to the Covid group, myofibroblasts showed significant upregulation in both the DM, HD, and HTN groups (**Figure 1C**). Conversely, no significant changes were observed in the subpopulations of immune cells, blood vessels, or other cell types (**Figures 1D–F**).

In another study, we used data from 45 healthy controls, 58 Covid-19 patients, and 64 Idiopathic Pulmonary Fibrosis (IPF) patients. Our findings revealed that compared to the healthy control group, Covid-19 patients were more prone to undergo endothelial-to-mesenchymal transition and epithelial-to-mesenchymal transition. Additionally, there was a significant increase in the proportion of myofibroblasts, which is considered a key factor in Covid-19-induced pulmonary fibrosis (24). Furthermore, this article focuses on the analysis of the pulmonary conditions in COVID-19 patients with diabetes, hypertension.

We further examined the differential expression of genes in the fibroblast group compared to the Covid group, which revealed decreased activity of DEGs specifically associated with myofibroblasts (**Figure 1G**, **sFigures 1B–F**). To gain more insight into the lung function within the fibroblast subpopulation, we applied a scoring method using scissor analysis (**Figures 1H, I**), which identifies cell subpopulations highly associated with a specific clinical phenotype based on bulk clinical data in scRNA-seq analysis (25). This analysis showed that the proportion of cells associated with poor lung function was significantly increased in the DM, HTN, and HD groups compared with the Covid group (**Figures 1J, K**). Subsequently, we isolated cells associated with poor lung function and performed differential expression analysis by comparing them with cells associated with good lung function (**Figure 1L**). This analysis revealed DEGs, and further gene set enrichment analysis (GSEA) demonstrated greater enrichment of fibrosis-related signaling pathways, such as TGF-β and WNT signaling (**Figure 1M**, **sFigures 2A–D**).

### 3.2 Fibroblasts are the major cellular subpopulation responsible for the upregulation of diabetic hypertensive drugs after SARS-CoV-2 infection

**Figure 2:**
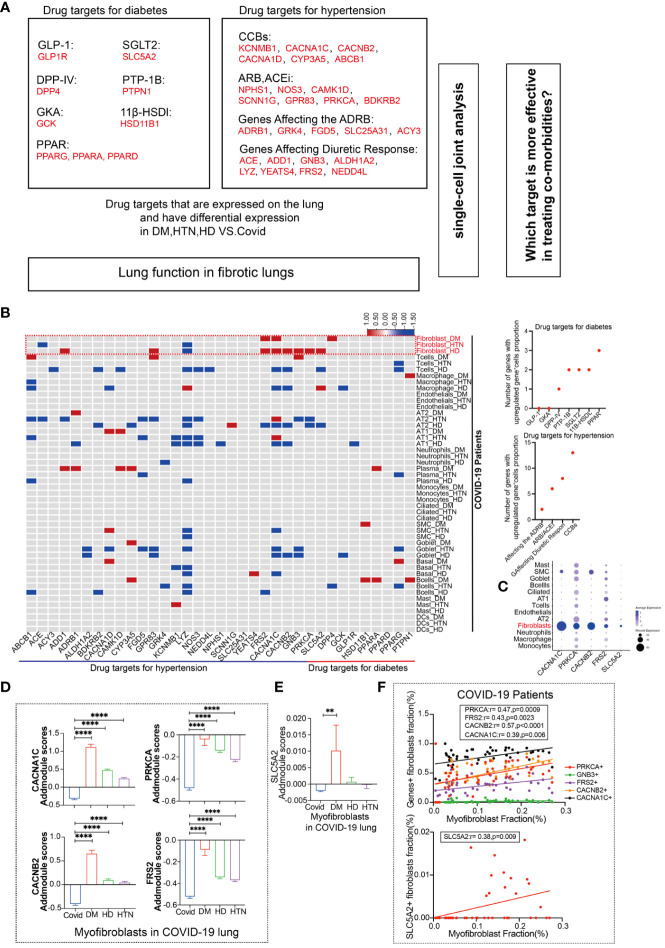
Fibroblasts have more targets for hypertension, diabetes drug response. **(A)** Schematic diagram showing the workflow for the analysis of common targets of common diabetes and hypertension drugs expressed in the lungs. Among the hypertensive drug target genes include Calcium channel blockers (CCBs): KCNMB1, CACNA1C, CACNB2, CACNA1D, CYP3A5, CYP3A4, ABCB1; Angiotensin-II Receptor Blockers (ARB) and Angiotensin-Converting Enzyme Inhibitors (ACEi): NPHS1, NOS3, CAMK1D, SCNN1G, GPR83, PRKCA, BDKRB2; Polymorphisms in Genes Affecting the Adrenergic Receptor Blocker Response : ADRB1, GRK4, FGD5, SLC25A31, ACY3; Polymorphisms in Genes Affecting Diuretic Response: ACE, ADD1, GNB3. ALDH1A2, LYZ, YEATS4, FRS2, NEDD4L. Diabetes drug target genes include: Glucagon-like peptide-1 (GLP-1): GLP1R; Novel glucose-sodium co-transport protein 2 (SGLT2). SLC5A2; Dipeptidyl peptidase IV (DPP-IV): DPP4; Peroxisome proliferator-activated receptor (PPAR): PPARG, PPARA, PPARB; Protein tyrosine phosphatase-1B (PTP-1B): PTPN1; Glucokinase agonists (GKA): GCK; 11β-hydroxysteroid dehydrogenase type 1 (11β-HSDl) inhibitors, including HSD11B1. **(B)** The heatmap presents the results of a meta-analysis showing the differences in the percentage of positive cells for hypertension and diabetes drug target genes in various cell subpopulations (Fibroblasts, T cells, Macrophages, Endothelial cells, AT2, AT1, Neutrophils, Plasma, Monocytes, Ciliated, SMC, Goblet, Basal, B cells, Mast, DCs) in lung tissues of the four patient groups (DM, HTN, HD) compared to the Control group. The dot plots display the number of upregulated positive cells in the percentage of drug-targeted genes across different cell types within hypertension and diabetes groups. **(C)** The DOT plot shows the expression of hypertension target genes (CACNA1C, CACNB2, PRKCA, FRS2) and diabetes target gene SLC5A2 in all lung cell subtypes. **(D)** The bar graph represents the Addmodule Scores of hypertension target genes (CACNA1C, CACNB2, PRKCA, FRS2) in myofibroblasts of patients in the Control, DM, HTN, and HD groups. The scores were significantly higher in the DM, HTN, and HD groups compared to the Control group. **(E)** The bar graph shows the Addmodule Scores of the diabetes target gene SLC5A2 in myofibroblasts of patients in the Control, DM, HTN, and HD groups. The scores were elevated in the DM, HTN, and HD groups compared to the Control group. **(F)** The correlation analysis demonstrates the positive correlation between the myofibroblast ratio and hypertension and diabetes target genes using the Pearson test. Four hypertension drug-related target genes and one diabetes target gene showed positive correlations with myofibroblasts. **P < 0.01, ****P < 0.0001.

In our analysis, we investigated the expression of common targets of diabetic and hypertensive drugs in the lungs of patients following SARS-CoV-2 infection (**Figure 2A**). The red color in the figure represents genes that were statistically upregulated, indicating that the targets of diabetic and hypertensive drugs were among the most altered in fibroblasts. Specifically, we observed that the diabetic target gene SLC5A2 was upregulated in fibroblasts, while hypertensive target genes CACNA1C, PRKCA, CACNB2, and FRS2 were also upregulated in fibroblasts (**Figure 2B**). Additionally, we categorized and identified genes that were both classified as drug targets in diabetes and hypertension and upregulated in our analysis (**Figure 2B**, **sFigures 3A**). Upon further analysis of the expression levels of these genes across all lung cell subtypes, we found that they were indeed highest in myofibroblasts (**Figure 2C**). Furthermore, we compared the expression of upregulated target genes specifically in diabetic myofibroblasts (**Figures 2D, E**). This analysis indicated that these target genes were indeed expressed at higher levels in diabetic myofibroblast cells. Finally, we identified the genes that showed a positive association with myofibroblast cells (**Figure 2F**). These findings suggest that the expression of some targets of diabetic hypertensive drugs may be dysregulated in the lungs of patients after SARS-CoV-2 infection and that myofibroblasts are the major cellular subpopulation responsible for the upregulation of diabetic hypertensive drugs after SARS-CoV-2 infection. This may promote the occurrence of pulmonary fibrosis in patients with COVID-19 combined with hypertension and diabetes.

### 3.3 Association of entry factors with fibroblast alterations and correlation with myofibroblasts and upregulated drug targets

**Figure 3:**
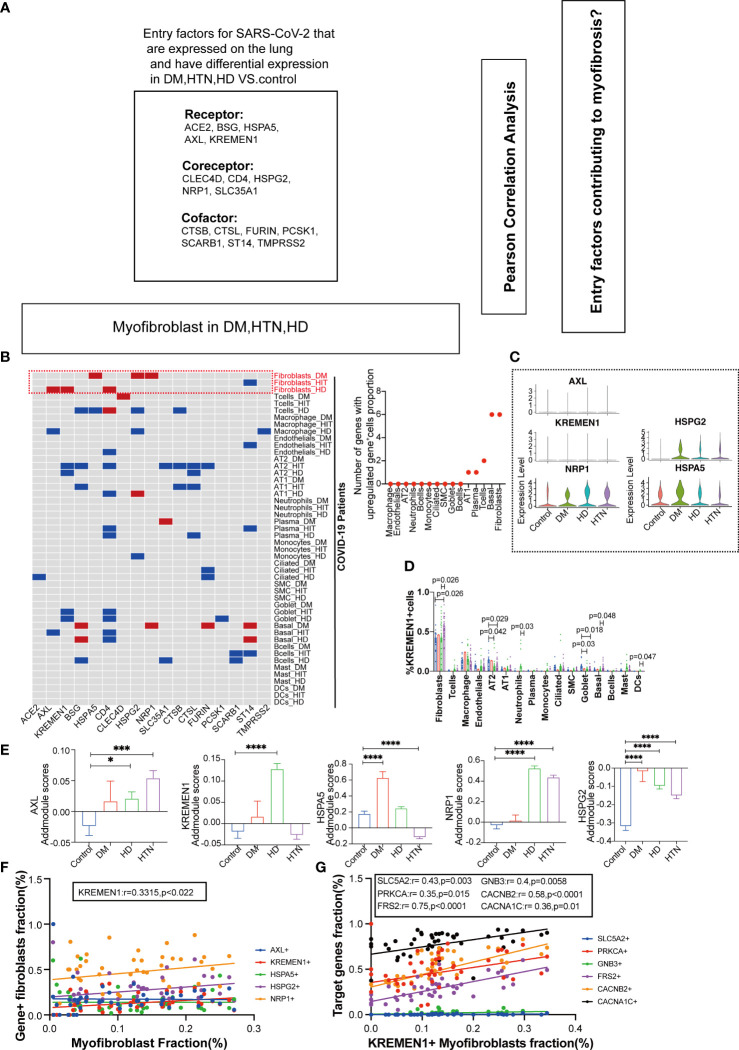
Association of Entry Factors with Fibroblast Alterations and Correlation with Myofibroblasts and Upregulated Drug Targets. **(A)** Schematic diagram showing the workflow for the analysis of entry factors expressed in the lung. Among the entry factors Receptor: ACE2, BSG, HSPA5, AXL, KREMEN1; Cofactor: CTSB, CTSL, FURIN, PCSK1, SCARB1, ST14, TMPRSS2; Coreceptor: CLEC4D, CD4, HSPG2, NRP1, SLC35A1. **(B)** Heatmap showing the meta-analysis of the differences in the percentage of entry factors positive cells in each cell subpopulation (Fibroblasts, T cells, Macrophages, Endothelials, AT2, AT1, Neutrophils, Plasma, Monocytes, Ciliated, SMC, Goblet, Basal, Bcells, Mast, DCs) in the lung tissues of the four groups of patients (DM, HTN, HD) vs. the Control group. Meta-analysis of differences in the percentage of cells positive for the entry factors in Ciliated, SMC, Goblet, Basal, Bcells, Mast, DCs. (Red indicates up-regulation, blue indicates down-regulation, and gray indicates no significant change). Dot plots show the number of up-regulated invasion target gene positive cell percentage in different cell subpopulations, respectively. **(C)** Violin plots showing the expression levels of invasion-associated genes (AXL, KREMEN1, HSPA5, NRP1, HSPG2) in four groups of diseases. **(D)** The bars show the percentage of KREMEN1-positive cells in each cell subpopulation, which was found to be significantly elevated in fibroblasts. **(E)** The bar graphs show the addmodule scores of invasion-associated genes (AXL, KREMEN1, HSPA5, NRP1, HSPG2) in myofibroblasts of patients with four diseases Control group (blue), DM group (red), HTN group (green), and HD group (purple). Scores were elevated in the DM, HTN, and HD groups relative to the Control group. (*P < 0.05, ***P < 0.001,****P < 0.0001). **(F)** Correlation analysis of myofibroblast fraction with new crown entry factors (Pearson test), showing KREMEN1 is positively correlated with myofibroblasts. **(G)** Correlation analysis of hypertension-targeted genes (CACNA1C, CACNB2, PRKCA, FRS2) and diabetes-targeted gene (SLC5A2) with entry factors KREMEN1 (Pearson test), showing KREMEN1 is positively correlated with drug target genes.

In our investigation of the effects of entry factors on the lungs in patients with the combined presence of diabetes, hypertension, and SARS-CoV-2 infection, we focused on 17 genes related to receptors, coreceptors, and cofactors (**Figure 3A**). By comparing the expression levels of the entry factors across all cell subpopulations in the three diseases with the Covid group, we observed that fibroblasts exhibited the most significant alterations (**Figure 3B**, **sFigures 4A–D**).We further evaluated the impact of the entry factors in two ways. Firstly, we extracted the proportions of cells expressing these factors, indicating an increase in diabetic and hypertensive fibroblasts (**Figures 3C, D**). Additionally, we performed scoring of several entry factors using the “Addmodule Score” method, suggesting varying degrees of upregulation in the three diseases (**Figure 3E**). Subsequently, we conducted correlation analysis between the last five entry factors and other variables. Interestingly, we found that the receptor gene KREMEN1 exhibited a positive correlation with myofibroblasts (**Figure 3F**). Furthermore, KREMEN1 showed a positive correlation with the upregulated drug targets (**Figure 3G**, **sFigures 4E–G**). These findings suggest that KREMEN1 displayed a positive correlation with myofibroblasts. This suggests a potential role of KREMEN1 in the fibrotic process. Additionally, KREMEN1 showed a positive correlation with upregulated drug targets, highlighting its involvement in disease-related pathways and potential therapeutic implications.

### 3.4 Activated endothelial cells may cause aggregation of fibroblasts

**Figure 4:**
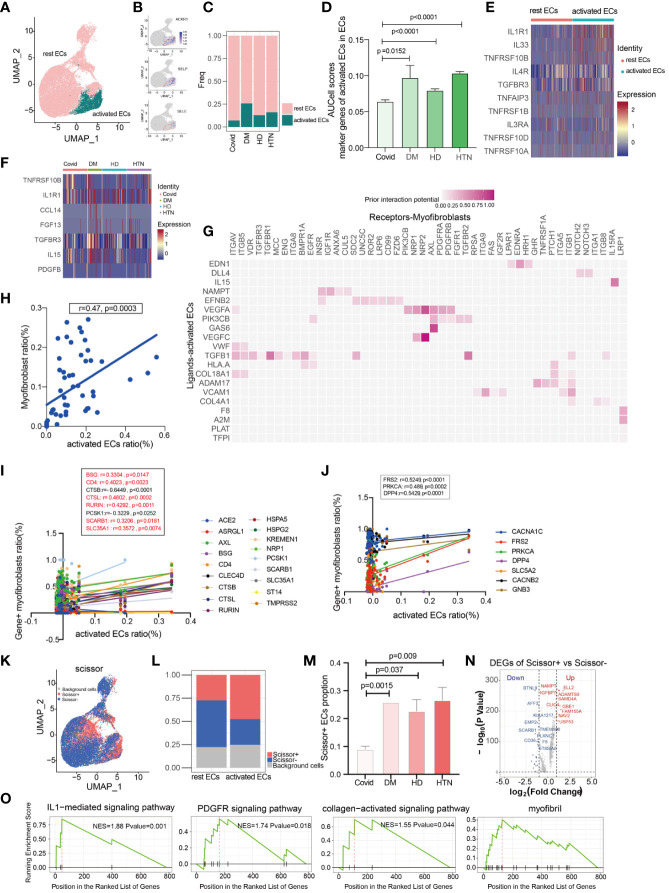
Activated endothelial cells may cause aggregation of fibroblasts. **(A)** UMAP visualization of endothelial cells, colored by celltype compartment. **(B)** UMAP shows the marker of activated ECs, mainly focusing on ACKR1, SELP, SELE. **(C, D)** Bar graphs demonstrate the proportion of endothelial cell subsets in the four disease groups; the DM, HTN and HD groups had significantly higher proportions of activated endothelial cells than the Control group. **(E)** Heatmap showing a comparison of major cytokine expression in endothelial cell subpopulations, indicating that activated endothelial cells are enriched in more cytokines (IL1R1, IL33, TNFRSF10B, IL4R, TGFBR3, TNFAIP3, TNFRSF1B, IL3RA, TNFRSF10D, TNFRSF10A) compared to other groups. **(F)** Heatmap showing the cytokines that were significantly elevated in the activated endothelial cells in the DM, HTN, and HD groups relative to the Control group: CCL14, FGF13, TGFBR3, IL15, and PDGFB in the DM group; TNFRSF10B in the HTN group; and TNFRSF10B and IL1R1 in the HD group. **(G)** NicheNet analysis showing ligand maps of activated endothelial cells with myofibroblast bodies. Darker colors in the matrix represent greater ligand-receptor interaction capacity. **(H)** Correlation analysis of myofibroblast with activated endothelial cells (Pearson test), showing activated endothelial cells is positively correlated with myofibroblasts. **(I)** Correlation analysis of SARS-CoV-2 entry factors with activated endothelial cells (Pearson test), showing that activated endothelial cells is positively correlated with the entry factors (BSG, CD4, CTSL, RURIN, SCARB1, SLC35A1). **(J)** Correlation analysis of hypertension/diabetes drug target genes with activated endothelial cells (Pearson test), showing activated endothelial cells is positively correlated with hypertension/diabetes drug target genes (FRS2, PRKCA, DPP4). **(K)** Scissor analysis relating lung function phenotypic information from bulk-RNA data of IPF patients from GEO with endothelial cells of COVID-19. UMAP plots indicate the position of cells normal lung function (blue) or low lung function (red) associated with lung function. **(L)** Bar graph showing the proportion of Scissor+, Scissor-, and background cells in rest ECs versus activated ECs. **(M)** Bar graph showing the cell proportion of Scissor+ endothelial cells in the four disease groups, the cell proportion of Scissor+ fibroblasts was significantly higher in the DM, HTN and HD groups relative to the Control group and the difference was statistically significant. **(N)** Volcano plot showing Scissor+ cell vs. Scissor- cell DGEs. the number of differentially up-regulated genes is 413 and the number of down-regulated genes is 252. **(O)** The GSEA enrichment analysis of differential genes has revealed that the IL1-mediated signaling pathway, PDGFR signaling pathway, collagen-activated signaling pathway, and myofibril were significantly enriched in the Scissor+ endothelial cells when compared to scissor- endothelial cells. The NES statistic of the enriched pathway score is positive and indicates that the pathway gene set is enriched and is in front of the sorting sequence. Conversely, if the NES statistic is negative, it means that a certain functional gene set is enriched behind the sorting sequence. When the Pvalue is less than 0.05, it indicates that the results are statistically significant.

It is well-known that the pulmonary vasculature is a common target in hypertension and diabetes, particularly affecting vascular endothelial cells (26). In our study, we identified two subpopulations of endothelial cells: rest endothelial cells and activated endothelial cells (**Figure 4A**). Activated endothelial cells exhibited high expression levels of the atypical chemokine receptor 1 (ACKR1) and adhesion molecules SELE (E-selectin) and SELP (P-selectin) (**Figure 4B**) (27, 28). Notably, activated endothelial cells exhibited significant upregulation in both diabetes and hypertension (**Figures 4C, D**). To further characterize activated endothelial cells, we examined the expression of cytokines and fibrogenic factors. We found that cytokines were significantly upregulated in activated endothelial cells (**Figure 4E**), suggesting an increased inflammatory response. Fibrogenic factors were also significantly upregulated in activated endothelial cells (**Figure 4F**), implying their involvement in fibrosis development. Utilizing a ligand-receptor approach, we identified several myofibroblast-inducing factors, such as TGF-beta and VEGFC, that are highly expressed in activated endothelial cells (**Figure 4G**). Additionally, activated endothelial cells showed a positive correlation with myofibroblasts (**Figure 4H**, **sFigure 5**). We further explored the correlation between activated endothelial cells and entry factors, indicating that most of the entry factors were indeed correlated (**Figure 4I**). Moreover, there was a correlation with hypertension-diabetes targets (**Figure 4J**). Lastly, investigating changes in lung function, we found that activated endothelial cells was associated with worse lung function (**Figures 4K–M**). After calculating the differential expression of scissor + and scissor -, signaling pathways such as IL1, PDGFR, and collagen were suggested to be up-regulated after analysis by GSEA clustering (**Figures 4N, O**). These findings suggest that the activated endothelial cells play a role in the exacerbation of lung effects in patients with hypertension and diabetes following SARS-CoV-2 infection.

### 3.5 Nintedanib but not Pirfenidone is more useful in patients with underlying fibrotic lungs with three disease

**Figure 5:**
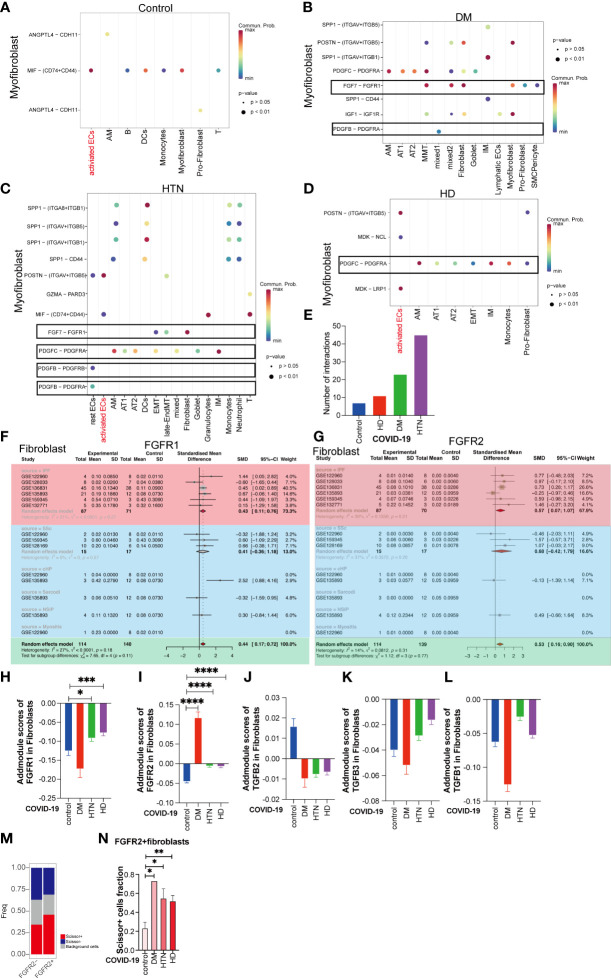
Nintedanib but not Pirfenidone is more useful in patients with underlying fibrotic lungs with three disease. **(A–D)** Dot plot demonstrating that in Control group, DM group, HTN group and HD group, the remaining cell subpopulations act as signal senders and can have cellular conversations with myofibroblasts via ligand-receptors. Among them, the gene pairs in Control group are ANGPTL4-CDH11, ANGPTL4-CDH11, MIF-(CD74+CD44); DM group: SPP1-(ITGAV+ITGB5), SPP1- (ITGAV+ITGB1), SPP1-CD44, POSTN- (ITGAV+ITGB5), IGF1-IGF1R, PDGFC- PDGFRA, PDGFB-PDGFRA, FGF7-FGFR1; HTN group: SPP1-(ITGA8+ITGB1), SPP1-(ITGAV+ITGB5), SPP1 -(ITGAV+ITGB1), SPP1-CD44, POSTN-(ITGAV+ITGB5), GZMA-PARD3, MIF-(CD74+CD44), FGF7 - FGFR1, PDGFC-PDGFRA, PDGFB - PDGFRA, PDGFB - PDGFRB; HD group: POSTN-(ITGAV+ITGB5), the MDK-NCL, PDGFC - PDGFRA, MDK-LRP1. **(E)** The bar graphs show the number of pairs of handheld ligands that produce cellular interactions with myofibroblasts in the four diseases and were found to be significantly higher in the disease groups. **(F, G)** Subgroup analysis of the difference in the proportion of Nintedanib -targeted gene-positive fibroblasts in pulmonary fibrosis versus healthy controls. Subgroup analysis of the difference in the proportion of Nintedanib -targeted gene-positive fibroblasts in pulmonary fibrosis versus healthy controls. the proportion of FGFR1, and FGFR2 gene-positive fibroblasts was significantly higher in ILD. **(H)** The bar graph illustrates the addmodule score of FGFR1 in fibroblasts of patients with four diseases: Control group (blue), DM group (red), HTN group (green), and HD group (purple). The scores of HTN group and HD group show an increase compared to the Control group (*P value<0.05, ***P value<0.001). **(I)** The bar graph displays the addmodule score of FGFR2 in fibroblasts of patients with four diseases: Control group (blue), DM group (red), HTN group (green), and HD group (purple). The scores of DM group, HTN group, and HD group show an increase compared to the Control group (****P value<0.0001). **(J–L)** The bar graph demonstrates the addmodule scores of TGFB1, TGFB2, and TGFB3 in fibroblasts of patients with four diseases: Control group (blue), DM group (red), HTN group (green), and HD group (purple). **(M)** The bar graph shows the proportion of Scissor+, Scissor-, and background cells in FGFR2- and FGFR2+ groups. The proportion of Scissor+ fibroblast cells increase in the FGFR2+ group relative to the FGFR2- group. **(N)** The bar graph indicates that among the FGFR2+ fibroblasts, the proportion of Scissor+ cells in the four disease groups is significantly higher than that in the Control group, DM group, HTN group, and HD group, and the difference is statistically significant. (*P < 0.05, **P < 0.01)

Based on the Cell Chat analysis, we observed that there are numerous receptor-ligand interactions between the targets of Pirfenidone and Nintedanib, specifically in the context of hypertension and diabetes (**Figures 5A–D**). Furthermore, the levels of these receptor-ligand interactions were progressively elevated during the onset of diabetes (**Figure 5E**). This led us to hypothesize that Pirfenidone and Nintedanib may have a therapeutic role in treating fibrosis sequelae caused by COVID-19, particularly in individuals with hypertension and diabetes. To identify the targets that are upregulated in fibroblasts, we incorporated five different fibrotic targets. Our analysis revealed that the main targets upregulated by Nintedanib were Fgfr1 and Fgfr2 (**Figures 5F, G**, **sFigures 6A–C**). After Addmodule Score analysis, Fgfr1 and Fgfr2 were increased in hypertension as well as hypertension-diabetes (**Figures 5H, I**), respectively, but no significant changes were seen in TGF-b1, TGF-b2, and TGF-b3, the targets of Pirfenidone (**Figures 5J–L**). We set scissor+ as poor lung function and scissor- as normal lung function, and a comparison of the two suggests that scissor+ ratios are upregulated in hypertension and diabetes. Also, scissor+ cell proportion was upregulated in hypertension, diabetes, and hypertension-diabetes groups versus Covid group (**Figures 5M, N**, **sFigure 6D**). Finally, we identified that the targets of Nintedanib, specifically Fgfr1 and Fgfr2, are indeed upregulated in individuals with hypertension and diabetes. This suggests that Nintedanib may have potential therapeutic benefits for targeting fibrosis in the lungs of patients with COVID-19 who also have comorbid hypertension and diabetes.

## 4 Discussion

We are the first single-cell meta-analysis of SARS-CoV-2 infection lungs of diabetes, hypertension, and hypertension-diabetes patients. We identify that lung function is worse in fibroblasts in all three diseases. We identified that lung function was worse in fibroblasts in all three diseases condition and identified that myofibroblast expression was elevated in diabetes, hypertension, and hypertension-diabetes patients, and identified five targets in diabetes, hypertension where myofibroblast was elevated, suggesting a better therapeutic regimen. For the entry factor of SARS-CoV-2, it was KREMEN1 rather than ACE2 that dominated its myofibroblast elevation, while finally identifying the antifibrotic agent, Nintedanib, as a possible target for diabetes, hypertension, and hypertension-diabetes in SARS-CoV-2 infection-induced fibrotic lungs.

The specific subtype of cell that is damaged by diabetes or hypertension is myofibroblast, and using fibrotic lung function, mapped to fibroblasts, we can see that patients with diabetes, hypertension, or diabetes-hypertension have worse lung function. Digging further into what causes the increased myofibroblast, we find that the target organ that is damaged by both diabetes and hypertension, the endothelial cells, has significantly higher activated endothelial cells. The major marker gene for endothelial cells, ACKR1, is now thought to be a gene associated with fibrosis (29), is also a marker gene for activated endothelial cells, and our current results suggest that in addition to increased myofibroblast, the expression of activated endothelial cells is also significantly increased, while becoming positively correlated with myofibroblast expression. This is because more and more studies are beginning to consider the lung as a “target organ” for diabetes or hypertension (30). This is not surprising, as pulmonary circulation is a major source of diabetes and hypertension (25). This is not surprising since the pulmonary circulation has the largest capillary network in the body and can accommodate the entire cardiac output (31). This is not surprising since the pulmonary circulation has the largest capillary network in the body and accommodates the entire cardiac output. Microvascular lesions in the lungs have been identified and have been described in autopsy studies and include thickening of the alveolar epithelium and capillary basement membranes (32). This structural abnormality is similar to those observed in the diabetic kidney and retina. Therefore, we suggest that activated endothelial cells may be a potential “bridge” leading to increased myofibroblast. By targeting endothelial cells, we may be able to slow down the increase in myofibroblast, thereby achieving the goal of slowing down lung function.

Interestingly, in our study we did not observe an increase in therapeutic targets in patients with hypertension alone, but in patients with comorbid hypertension-diabetesor diabetes mellitus, we observed an increase in targets in relation to each other. Positively associated with the increased targets was increased myofibroblast, suggesting that patients with combined hypertension-diabetes may be more impaired after SARS-CoV-2 infection. This may be related to more severe cumulative damage to blood vessels and stromal cells in hypertension-diabetesor diabetes mellitus (33, 34). Further study of these findings will allow us to better understand the effects of COVID-19 in patients with comorbid hypertension-diabetes and to search for more effective treatments. In this study, an inhibitor of the antidiabetic drug SGLT2 (sodium-glucose cotransporter-2/SLC5A2), which reduces renal reabsorption of glucose to lower blood glucose, could indirectly reduce lung infection in diabetic mice (35). However, a new clinical study was initiated in April 2020 to understand the substantial cardiorenal protective effects of SLGT2 inhibitors in reducing disease progression, complications, and all-cause mortality in hospitalized adult patients with a focus on those with COVID-19 (36, 37). Our study suggests that SLC5A2 is elevated in fibroblasts and positively correlates with myofibroblast, predicting that SLC5A2 is target of COVID-19 related-fibrosis.

According to the literature, host cell receptors play a key role in determining viral tropism and pathogenesis (38). However, little is known about the host receptors for SARS-CoV-2 other than ACE2 (39, 40). In our study, we did not observe upregulation of classical entry factors such as ACE2; instead, we found that the upregulation of KREMEN1 correlated with an increase in myofibroblast. This suggests that ACE2 alone cannot explain the multiorgan invasiveness of SARS-CoV-2 or the fact that ACE2 expression is not significantly altered after SARS-CoV-2 infection. Indeed, KREMEN1 plays a sufficiently important role in SARS-CoV-2 entry (41, 42), and we therefore suggest that KREMEN1 may act as an alternative functional receptor that plays an important role in ACE2-independent viral invasion. This also provides the explanation that diabetic and hypertensive patients may still be associated with increased myofibroblast in the absence of significant ACE2 elevation. In addition, our study shows that KREMEN1-positive cells have poorer lung function, providing us with a relevant basis. Taken together, our study reveals that KREMEN1 may be an important alternative receptor in SARS-CoV-2 infection, playing an important role in lung fibrosis and deterioration of lung function (41). Finally, by examining patients with the three diseases combined diabetes, hypertension, and hypertension-diabetes after SARS-CoV-2 infection, we propose that activated endothelial cells may be a potential bridge to fibrosis, as well as suggesting that the preference of anti-hypertensive drug targets and the influence of entry factors on the combined three diseases is not a traditional ACE2, while proposing that Nintedanib is a superior choice for late fibrotic sequelae.

Nintedanib as add-on therapy for pulmonary fibrosis after COVID-19 did not improve oxygenation or mortality, but improved the SpO2/FiO2 ratio in patients (43). The use of Nintedanib alongside treatment of COVID-19 pulmonary fibrosis showed a more pronounced improvement in lung CT severity score compared with Pirfenidone (44). Consistent with our findings of receptor upregulation of Nintedanib in fibroblasts. Noth et al. found that the incidence of major adverse cardiovascular events was similar between the nintedanib and placebo groups, both in patients with high and low cardiovascular risk (45). However, it should be noted that the most frequent adverse events associated with nintedanib use are diarrhea, decreased appetite, and vomiting (46). These adverse events may have an impact on the blood glucose and blood pressure of patients with diabetes. Therefore, close monitoring of blood glucose and blood pressure is recommended when using nintedanib.

It is important to acknowledge the limitations of our study. Firstly, we were unable to conduct *in vitro* or *in vivo* animal experiments to directly evaluate the preferential target receptor for SARS-CoV-2 infection. Secondly, we did not perform *in vitro* experiments to validate the impact of Nintedanib. Thirdly, the sample size in our study was limited, which prevented us from conducting a more detailed analysis of the impact of gender within our subgroup.

## 5 Conclusion

In conclusion, the analysis of single-cell data from the lungs of COVID-19 patients with comorbid diabetes and hypertension allowed us to assess their biological characteristics comprehensively and systematically. In this study, we found that vascular-related cells, especially activated endothelial cells, play an important role in the co-morbid process of diabetes, hypertension, and COVID-19. At the same time, we suggest that fibroblasts may be the ultimate “effector cells” that contribute to the deterioration of lung function. Based on these findings, we propose that activated endothelial cells may be a potential “bridge” to fibrosis and suggest preferred drug targets for treatment. In addition, we found that in the combination of these three diseases, the entry factor used by SARS-CoV-2 is not the traditional ACE2, but possibly KREMEN1, and finally, we suggest that Nintedanib may be a preferred therapeutic option for the late fibrotic sequelae. These findings provide important academic and clinical implications for better understanding and treating pulmonary complications due to combined diabetes and hypertension in patients with COVID-19.

## Data availability statement

The datasets presented in this study can be found in online repositories. The names of the repository/repositories and accession number(s) can be found in the article/**Supplementary Material**.

## Author contributions

XZ: Formal Analysis, Funding acquisition, Methodology, Project administration, Supervision, Visualization, Writing – original draft, Writing – review & editing. XD: Data curation, Project administration, Writing – review & editing. LiZ: Writing – review & editing. PW: Data curation, Formal Analysis, Software, Writing – review & editing. XT: Data curation, Formal Analysis, Investigation, Resources, Writing – original draft. YM: Data curation, Formal Analysis, Software, Writing – original draft. YSZ: Data curation, Formal Analysis, Software, Writing – original draft. YZ: Writing – review & editing. CM: Writing – review & editing. LaZ: Conceptualization, Funding acquisition, Project administration, Supervision, Writing – original draft, Writing – review & editing.
