# Supplementary material for: Single-cell RNA sequencing analysis of lung cells in COVID-19 patients with diabetes, hypertension, and comorbid diabetes-hypertension
Source: Front Endocrinol (Lausanne). 2023 Dec 8;14:1258646. doi: 10.3389/fendo.2023.1258646 (PMC10748394; doi:10.3389/fendo.2023.1258646)
Supplement: Supplementary file 3 [file DataSheet_1.pdf]

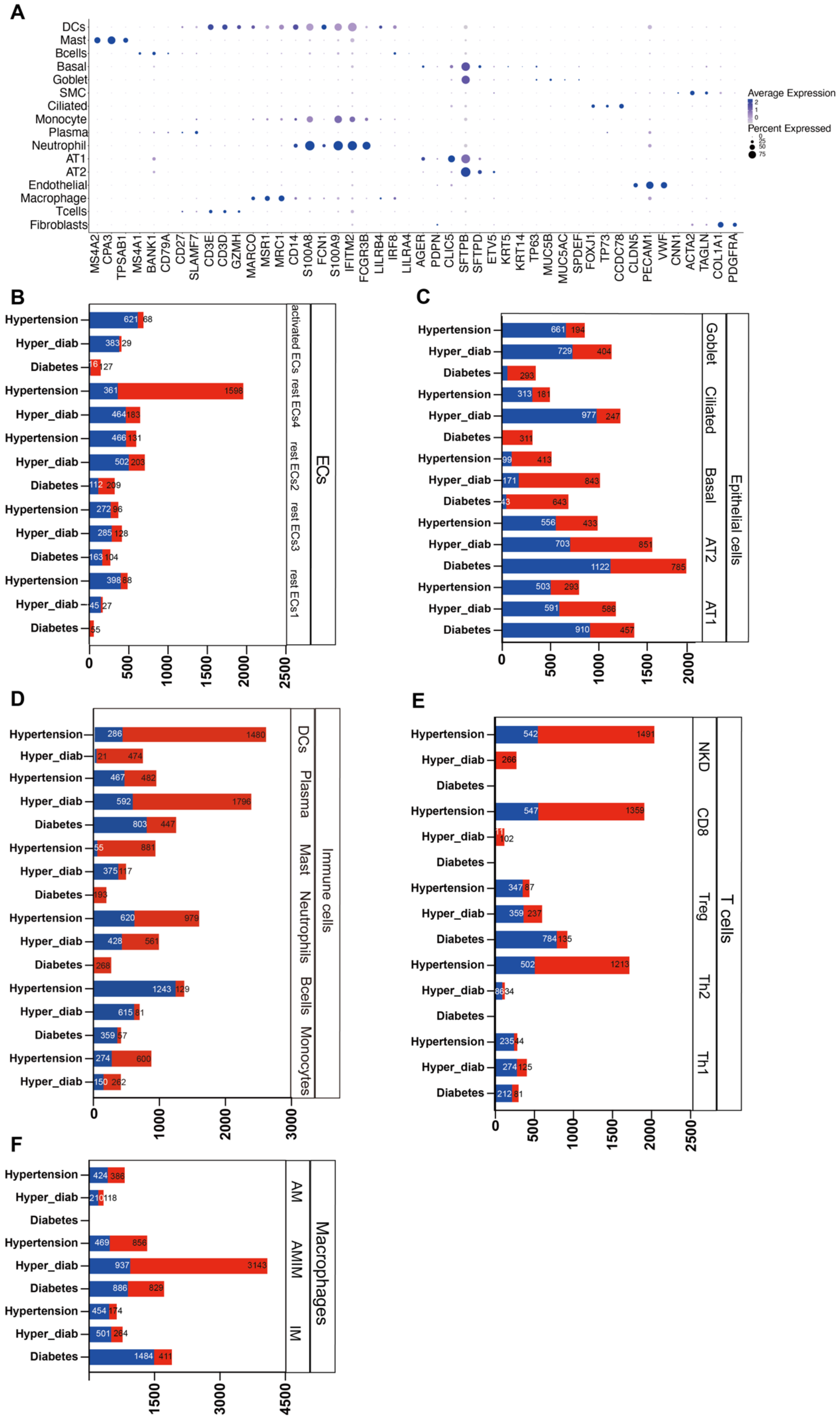
A
DCs
Mast
Bcells
Basal
Goblet
SMC
Ciliated
Monocyte
Plasma
Neutrophil
AT1
AT2
Endothelial
Macrophage
Tcells
Fibroblasts
MS4A2 CPA3 TPSAB1 MS4A1 BANK1 CD79A CD27 SLAMF7 CD3E CD3D GZMH MARCO MSR1 MRC1 CD14 S100A8 FCN1 S100A9 IFITM2 FCGR3B LILRB4 IRF8 LILRA4 AGER PDPN CLIC5 SFTPB SFTPD ETV5 KRT5 KRT14 TP63 MUC5B MUC5AC SPDEF FOXJ1 TP73 CCDC78 CLDN5 PECAM1 VWF CNN1 ACTA2 TAGLN COL1A1 PDGFRA
Average Expression
Percent Expressed
B
activated ECs
rest ECs4
rest ECs2
rest ECs3
rest ECs1
ECs
C
Goblet
Ciliated
Basal
AT2
AT1
Epithelial cells
D
DCs
Plasma
Mast
Neutrophils
Bcells
Monocytes
Immune cells
E
NKD
CD8
Treg
Th2
Th1
T cells
F
AM
AMIM
IM
Macrophages
Hypertension
Hyper_diab
Diabetes

sFigure1: related to Figure 1. Large differences in the transcriptome levels of lung cell subpopulations in COVID-19 patients with diabetes/hypertension

A. Dot chart shows the marker genes of each subpopulation of lung cells.

B. Bar graph exhibiting the number of differentially expressed genes (DEGs) in Endothelial cells from COVID-19 patients, comparing the DM, HTN, HD, and Covid groups, categorized by cell type (FDR < 0.05).

C. Bar graph exhibiting the number of differentially expressed genes (DEGs) in Epithelial cells from COVID-19 patients, comparing the DM, HTN, HD, and Covid groups, categorized by cell type (FDR < 0.05).

D. Bar graph exhibiting the number of differentially expressed genes (DEGs) in Immune cells from COVID-19 patients, comparing the DM, HTN, HD, and Covid groups, categorized by cell type (FDR < 0.05).

E. Bar graph exhibiting the number of differentially expressed genes (DEGs) in T cells from COVID-19 patients, comparing the DM, HTN, HD, and Covid groups, categorized by cell type (FDR < 0.05).

F. Bar graph exhibiting the number of differentially expressed genes (DEGs) in Macrophages from COVID-19 patients, comparing the DM, HTN, HD, and Covid groups, categorized by cell type (FDR < 0.05). （Red color represents upregulation, Blue color represents downregulation）

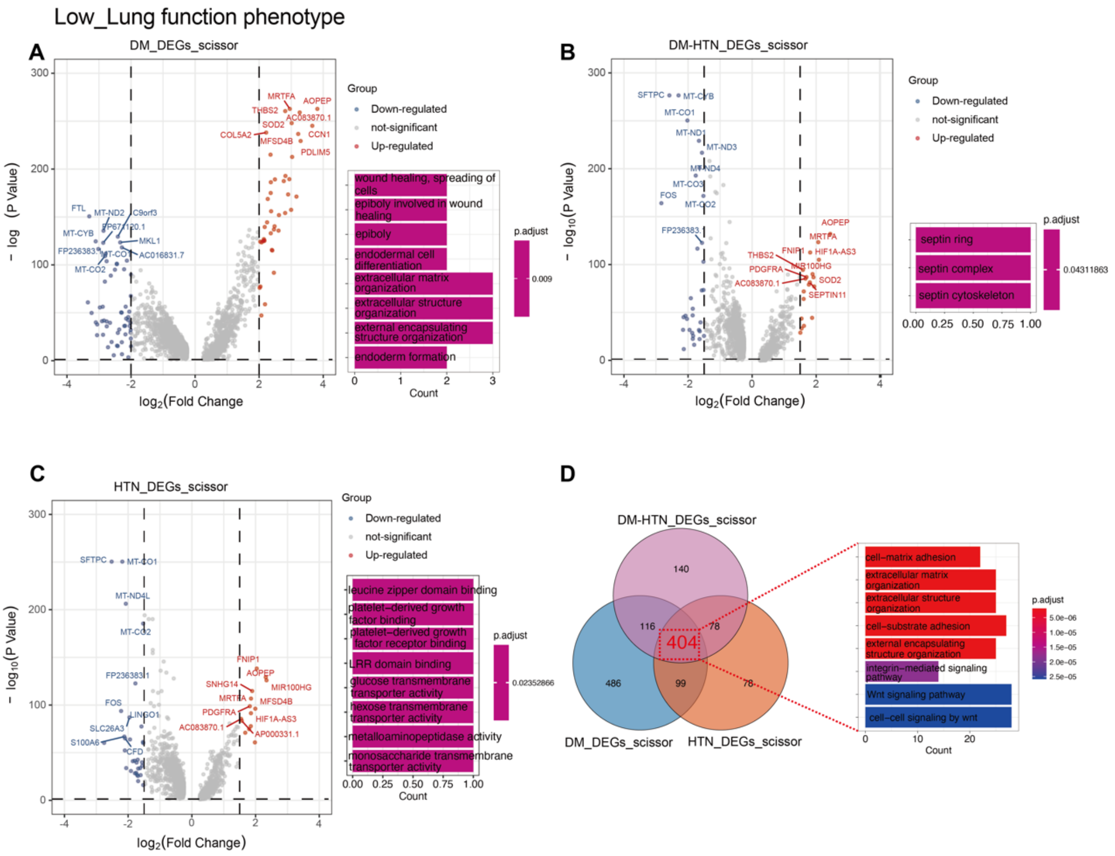

sFigure2: related to Figure1. Consistent Differential Expression and Increased Susceptibility to Pulmonary Fibrosis in COVID-19 Patients with Comorbidities of Diabetes and Hypertension.

A. Volcano plot displaying the differentially expressed genes (DEGs) between the DM group and the Covid group in Scissor+ cells. It indicates the count of upregulated and downregulated genes. Furthermore, bar graph presents the top 8 enriched signaling pathways for upregulated genes.

B. Volcano plot displaying the DEGs between the DM group and the HTN group in Scissor+ cells. It indicates the count of upregulated and downregulated genes. Bar graph presents the top 8 enriched signaling pathways for upregulated genes.

C. Volcano plot displaying the DEGs between the HTN group and the Covid group in Scissor+ cells. It indicates the count of upregulated and downregulated genes. Bar graph presents the top 8 enriched signaling pathways for upregulated genes.

D. The Venn diagram illustrates the intersection of upregulated differentially expressed genes (DEGs) between three groups (DM vs Covid, HTN vs Covid, DM vs HTN), consisting of a total of 404 genes. The bar graph displays the top 8 enriched signaling pathways for these 404

genes.

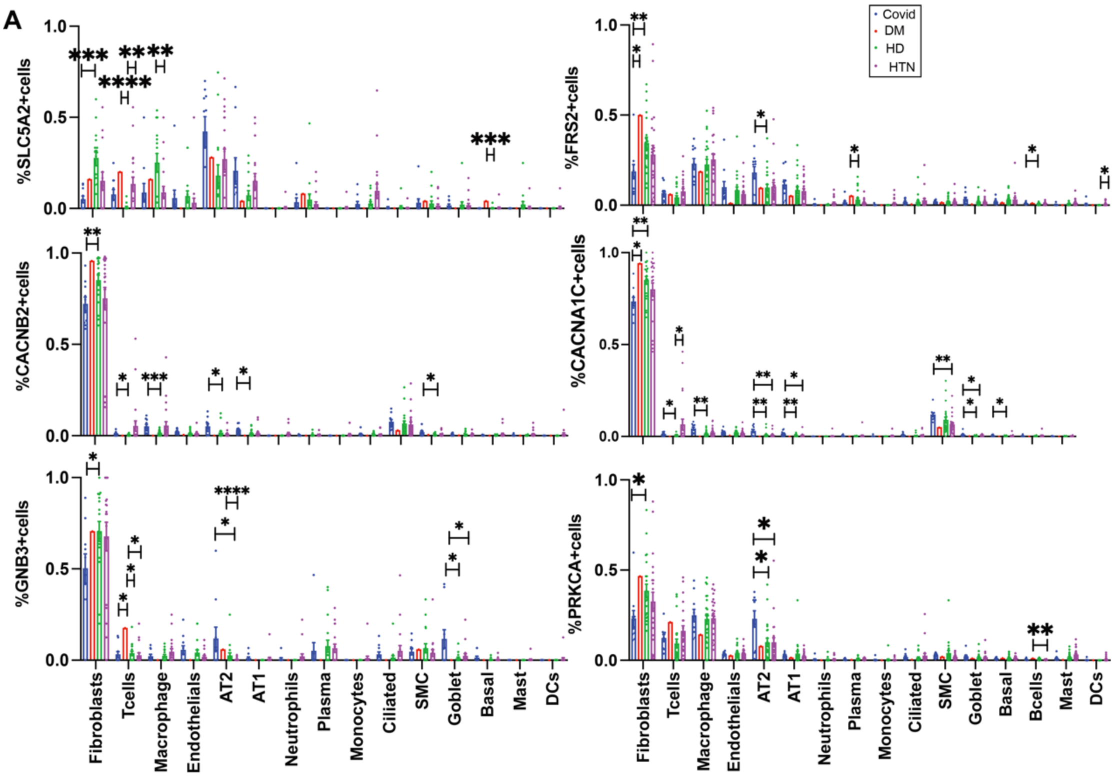

sFigure3: related to Figure2. Fibroblasts have more targets for hypertension, diabetes drug response

A. The bars show the percentage of gene (hypertension target genes (CACNA1C, CACNB2, PRKCA, FRS2) and diabetes target gene SLC5A2)-positive cells in each cell subpopulation, which was found to be significantly elevated in fibroblasts.

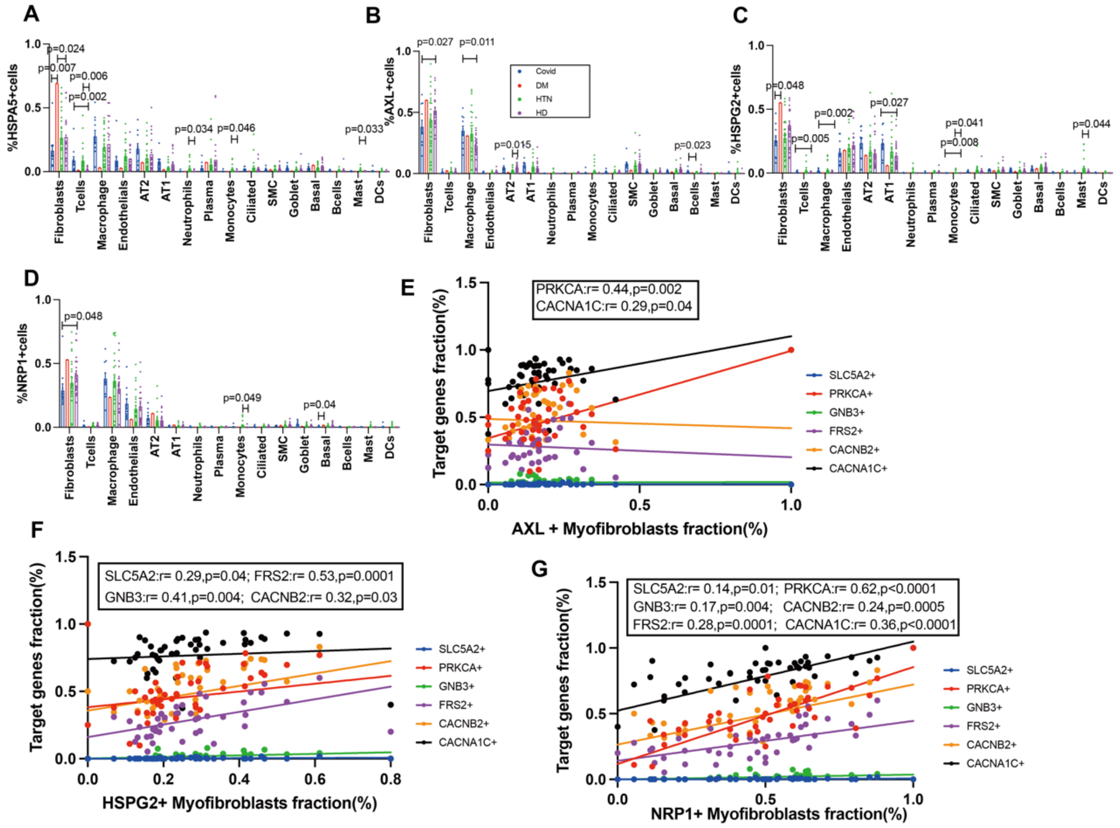

sFigure4: related to Figure3. Association of Entry Factors with Fibroblast Alterations and Correlation with Myofibroblasts and Upregulated Drug Targets

A. The bars show the percentage of HSPA5-positive cells in each cell subpopulation, which was found to be significantly elevated in fibroblasts.

B. The bars show the percentage of AXL-positive cells in each cell subpopulation, which was found to be significantly elevated in fibroblasts.

C. The bars show the percentage of HSPG2-positive cells in each cell subpopulation, which was found to be significantly elevated in fibroblasts.

D. The bars show the percentage of NRP1-positive cells in each cell subpopulation, which was found to be significantly elevated in fibroblasts.

E. Correlation analysis of hypertension-targeted genes (CACNA1C, CACNB2, PRKCA, FRS2) and diabetes-targeted gene (SLC5A2) with entry factors AXL (Pearson test), showing KREMEN1 is positively correlated with drug target genes.

F. Correlation analysis of hypertension-targeted genes (CACNA1C, CACNB2, PRKCA, FRS2) and diabetes-targeted gene (SLC5A2) with entry factors HSPG2 (Pearson test), showing

KREMEN1 is positively correlated with drug target genes.

G. Correlation analysis of hypertension-targeted genes (CACNA1C, CACNB2, PRKCA, FRS2) and diabetes-targeted gene (SLC5A2) with entry factors NRP1 (Pearson test), showing KREMEN1 is positively correlated with drug target genes.

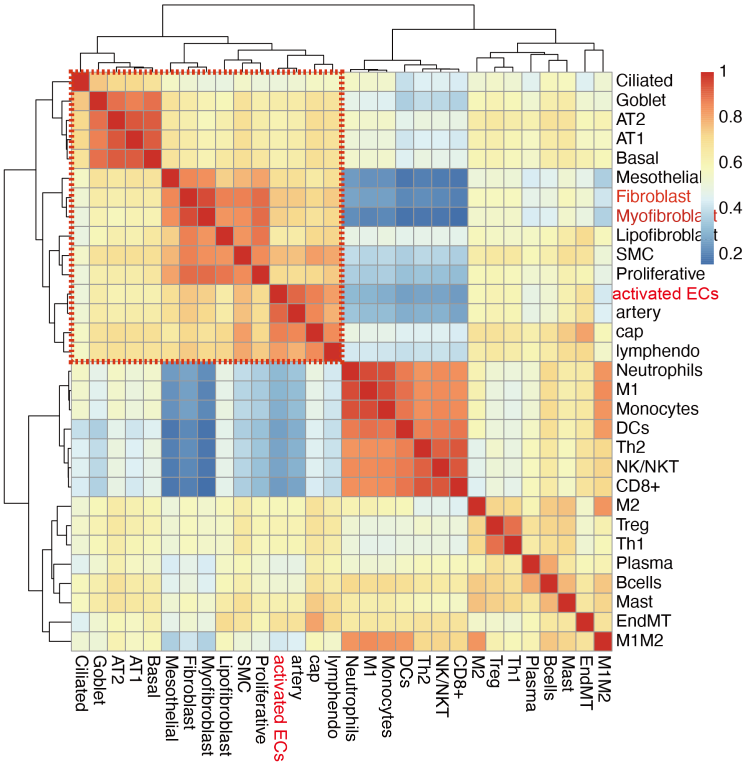

sFigure5: related to Figure4.

The heat map displays the correlation analysis among different cell subpopulations.

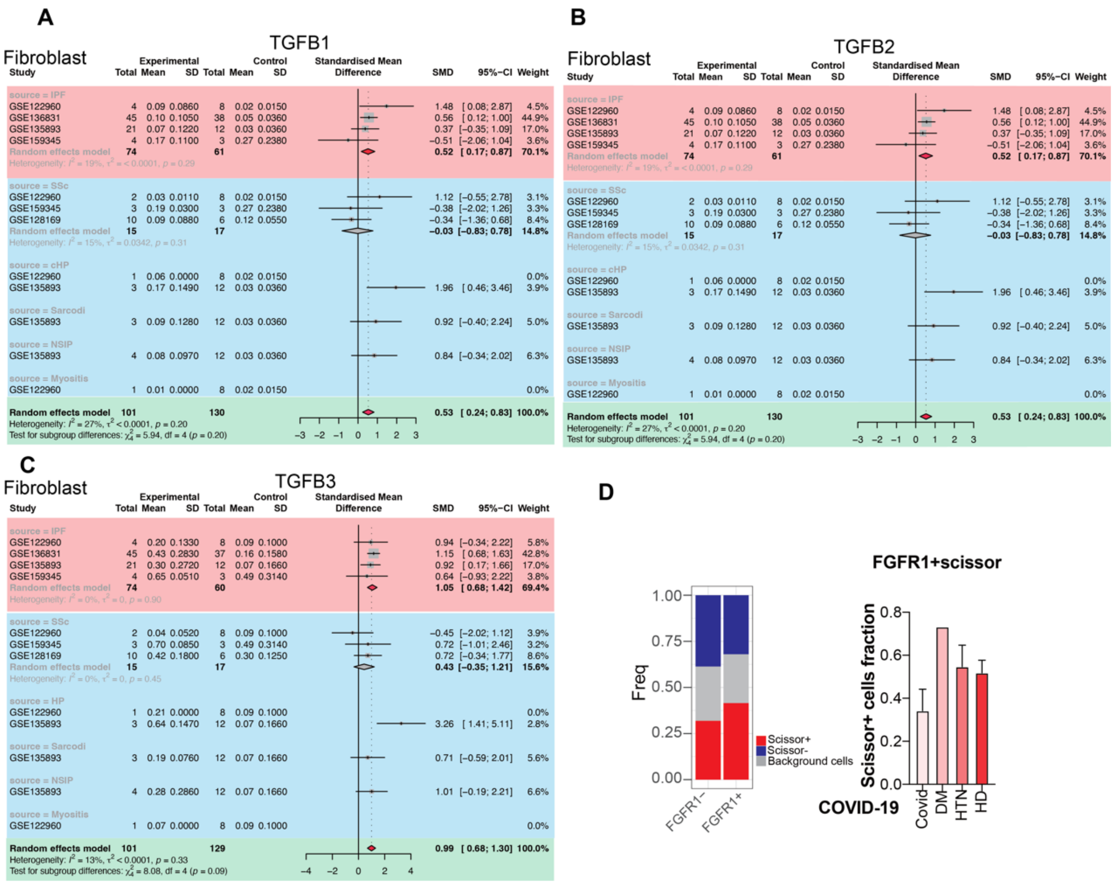

sFigure6: related to Figure5.

A-C. Subgroup analysis of the difference in the proportion of **Pirfenidone** -targeted gene-positive fibroblasts in pulmonary fibrosis versus healthy controls. The proportion of TGFB1, TGFB2, and TGFB 3 gene-positive fibroblasts was significantly higher in ILD.

D. The bar graph shows the proportion of Scissor+, Scissor-, and background cells in FGFR1- and FGFR1+ groups. The bar graph indicates that among the FGFR1+ fibroblasts, the proportion of Scissor+ cells in the three disease groups is higher than that in the Covid group, DM group, HTN group, and HD group, but the difference is not statistically significant.
